# Supplementary material for: Association between Mother’s Education and Infant and Young Child Feeding Practices in South Asia
Source: Nutrients. 2022 Apr 5;14(7):1514. doi: 10.3390/nu14071514 (PMC9003257; doi:10.3390/nu14071514)
Supplement: Supplementary file 1 [file nutrients-14-01514-s001.zip › nutrients-1622726-supplementary.pdf]

Table S1: Socio-demographic characteristics of the study participants

Characteristics	All countries (2015-2018)	Afghanistan (2015¥)	Bangladesh (2017-2018¥)	India (2015-2016¥)	Maldives (2016-2017¥)	Nepal (2016¥)	Pakistan (2017-2018¥)
	**n (%) n=120830**	**n (%) n=11762**	**n (%) n=3411**	**n (%) n=97935**	**n (%) n=1147**	**n (%) n=1919**	**n (%) n=4656**
**Mother's age**							
15-24 years	52217 (45.6)	4293 (37.6)	1883 (55.1)	43310 (47.0)	258 (20.1)	1034 (52.6)	1439 (31.6)
25-34 years	58794 (47.3)	5454 (44.8)	1328 (39.3)	48028 (47.5)	704 (61.9)	786 (42.2)	2494 (54.5)
≥35 years	9819 (7.1)	2015 (17.7)	200 (5.6)	6597 (6.7)	185 (18.1)	99 (5.2)	723 (13.9)
**Mother's education**							
<Secondary	57216 (46.1)	10751 (89.3)	1174 (34.0)	41308 (40.8)	190 (16.4)	900 (48.7)	2893 (63.1)
≥Secondary	63614 (53.9)	1011 (10.7)	2237 (66.0)	56627 (59.2)	957 (83.6)	1019 (51.3)	1763 (36.9)
**Child sex**							
Male	63146 (52.3)	6111 (52.0)	1769 (52.0)	51299 (52.5)	574 (59.6)	1038 (53.8)	2355 (50.3)
Female	57684 (47.7)	5651 (48.0)	1642 (48.0)	46636 (47.5)	573 (50.4)	881 (46.2)	2301 (49.7)
**Child age, mean (sd)**	11.32 (6.6)	10.6 (6.6)	11.2 (6.9)	11.4 (6.6)	11.7 (6.8)	11.8 (6.8)	10.8 (6.6)
**Place of residence**							
Urban	30159 (27.8)	2920 (24.2)	1167 (26.7)	22790 (27.5)	114 (9.9)	1089 (53.6)	2079 (33.1)
Rural	90671 (72.2)	8842 (75.8)	2244 (73.3)	75145 (72.6)	1033 (90.1)	830 (46.5)	2577 (67.0)
**Wealth Index**							
Poorest	29792 (23.7)	2019 (17.8)	724 (20.5)	25225 (24.8)	311 (27.1)	486 (21.0)	1027 (21.5)
Poorer	28187 (21.7)	2667 (20.0)	712 (21)	23044 (22.0)	327 (28.5)	407 (21.2)	1030 (19.1)
Middle	24750 (20.4)	2676 (21.5)	627 (19.2)	19765 (20.2)	328 (28.6)	413 (23.0)	941 (21.5)
Richer	20959 (18.6)	2654 (21.5)	684 (20.5)	16336 (18.1)	120 (10.5)	371 (20.5)	794 (18.6)
Richest	17142 (15.6)	1746 (19.3)	664 (18.8)	13565 (14.8)	61 (5.3)	242 (14.4)	864 (19.3)

¥Survey year

Table S2: Prevalence of IYCF practices among South Asian countries

IYCF Indicators	Pooled data	Afghanistan	Bangladesh	India	Maldives	Nepal	Pakistan
	**% (n)**	**% (n)**	**% (n)**	**% (n)**	**% (n)**	**% (n)**	**% (n)**
Early initiation of breastfeeding	52548 (45.4) N=115776	5620 (42.1) N=11433	2038 (60.4) N=3369	41711 (43.4) N=93514	798 (68.2) N=1121	1102 (55.6) N=1900	1279 (20.8) N=4439
Exclusive Breastfeeding under 6 months	16068 (53.9) N=29838	1659 (41.9) N=3331	605 (64.7) N=937	12662 (52.9) N=23547	170 (63.3) N=276	311 (66.5) N=452	661 (45.7) N=1295
Continued Breastfeeding at 1 year	17772 (82.8) N=21470	1983 (75.8) N=2578	536 (92.7) N=575	14127 (83.2) N=16198	174 (76.0) N=206	336 (94.7) N=354	616 (67.2) N=859
Introduction of Solid-semi-solid or soft foods	8415 (50.2) N=16778	921 (58.2) N=1646	287 (69.0) N=401	6158 (44.1) N=13706	111 (81.6) N=132	175 (78.5) N=228	379 (56.3) N=665
Minimum dietary diversity	19918 (21.9) N=90845	1673 (23.1) N=8373	940 (37.7) N=2472	15361 (20.7) N=74304	617 (71.4) N=869	671 (44.7) N=1467	656 (18.5) N=3360
Minimum meal frequency	36281 (39.9) N=90281	4185 (49.1) N=8431	1901 (79.1) N=2367	26640 (34.6) N=74388	562 (65.3) N=871	973 (70.0) N=1400	2020 (57.9) N=3361
Minimum acceptable diet	11622 (12.8) N=90992	1227 (16.6) N=8431	873 (34.8) N=2474	7975 (10.7) N=74388	439 (52.1) N=871	507 (34.2) N=1467	598 (16.5) N=3361

Consumption of iron-rich or iron fortified foods	18705 (21.4) N=87552	2202 (30.4) N=8022	1642 (68.0) N=2401	12583 (17.0) N=71647	584 (69.5) N=845	489 (34.5) N=1436	1205 (37.0) N=3201

n= numerators, N= Denominators of the respective indicators

Table S3: Bivariate association of IYCF indicators and mother's education

Indicators	Pooled		Afghanistan		Bangladesh		India		Maldives		Nepal		Pakistan	
	**<Sec**	**≥Sec**	**<Sec**	**≥Sec**	**<Sec**	**≥Sec**	**<Sec**	**≥Sec**	**<Sec**	**≥Sec**	**<Sec**	**≥Sec**	**<Sec**	**≥Sec**
EIBF (%)	43.6	47.0	41.9	43.7	65.2	57.9	39.7	46.0	71.8	67.5	53.1	58.0	22.1	18.5
	P<0.001		P=0.630		P<0.001		P<0.001		P=0.367		P=0.129		P=0.110	
EBF (%)	52	55.5	42.7	35.0	63.3	65.4	51.2	54.0	54.8	65.1	68.2	65.1	50.3	37.3
	P<0.001		P=0.155		P=0.570		P=0.003		P=0.306		P=0.544		P=0.002	
CBF (%)	82.9	82.7	75.2	80.6	91.4	93.4	84.9	82.0	85.6	74.0	92.5	96.8	71.8	59.8
	P=0.598		P=0.279		P=0.383		P<0.001		P=0.390		P=0.123		P=0.027	
ISSSF (%)	46.3	53.7	58.4	56.5	60.7	67.9	38.0	48.3	71.6	83.4	75.1	80.2	48.0	71.0
	P<0.001		P=0.818		P=0.238		P<0.001		P=0.201		P=0.398		P<0.001	
MDD (%)	17.3	26.1	21.9	33.1	27.3	42.8	15.6	24.3	59.8	73.6	35.3	54.0	12.1	29.2
	P<0.001		P=0.029		P<0.001		P<0.001		P=0.017		P<0.001		P<0.001	
MMF (%)	38.2	41.5	49.0	49.1	76.8	80.2	31.5	36.8	54.3	67.4	65.1	74.9	52.4	67.0
	P<0.001		P=0.054		P=0.102		P<0.001		P=0.022		P<0.001		P<0.001	
MAD (%)	10.0	15.3	15.7	23.5	25.0	39.6	7.8	12.7	39.5	54.4	25.6	42.7	10.9	25.9
	P<0.001		P=0.054		P<0.001		P<0.001		P=0.009		P<0.001		P<0.001	
CIRF (%)	19.1	23.4	29.5	37.4	59.1	69.5	14.1	19.0	69.0	69.6	29.2	39.7	29.5	49.2
	P<0.001		P=0.060		P<0.001		P<0.001		P=0.910		P<0.001		P<0.001	

Sec=secondary; P-values were generated from adjusted Wald test. EIBF= Early initiation of breastfeeding, EBF= Exclusive breastfeeding, CBF= Continued breastfeeding at 1 year, ISSSF=Introduction of solid semi-solid and soft foods, MDD= Minimum dietary diversity, MMF=Minimum meal frequency, MAD= Minimum acceptable diet, CIRF=Consumption of iron-rich or iron-fortified foods.

Table S4: Sensitivity analysis of the association of IYCF and maternal education (years of schooling)

IYCF Indicators	All countries (Pooled data)		Afghanistan		Bangladesh		India		Maldives		Nepal		Pakistan	
	**AOR (95% CI)**	**p-value**	**AOR (95% CI)**	**p-value**	**AOR (95% CI)**	**p-value**	**AOR (95% CI)**	**p-value**	**AOR (95% CI)**	**p-value**	**AOR (95% CI)**	**p-value**	**AOR (95% CI)**	**p-value**
**EIBF**														
<Secondary	Ref.		Ref.		Ref.		Ref.		Ref.		Ref.		Ref.	
≥Secondary	1.01 (1.005, 1.011)	<0.001	1.01 (1.00, 1.02)	0.134	0.97 (0.95, 0.99)	0.042	1.009 (1.005, 1.012)	<0.001	0.96 (0.91, 1.01)	0.133	1.02 (0.99, 1.04)	0.261	0.99 (0.97, 1.01)	0.176
**EBF**														
<Secondary	Ref.		Ref.		Ref.		Ref.		Ref.		Ref.		Ref.	
≥Secondary	1.02 (1.01, 1.24)	<0.001	0.98 (0.96, 1.01)	0.138	1.02 (0.98, 1.07)	0.367	1.03 (1.02, 1.032)	<0.001	1.06 (0.97, 1.17)	0.213	1.02 (0.96, 1.08)	0.494	0.95 (0.92, 0.98)	<0.001
**CBF**														
<Secondary	Ref.		Ref.		Ref.		Ref.		Ref.		Ref.		Ref.	
≥Secondary	1.04 (0.96, 1.14)	0.328	1.02 (0.98, 1.05)	0.387	1.06 (0.95, 1.18)	0.291	1.003 (0.993, 1.01)	0.622	1.00 (0.87, 1.15)	0.587	1.10 (0.97, 1.25)	0.144	0.99 (0.96, 1.03)	0.579
**ISSSF**														
<Secondary	Ref.		Ref.		Ref.		Ref.		Ref.		Ref.		Ref.	
≥Secondary	1.04 (1.03, 1.05)	<0.001	0.99 (0.95, 1.02)	0.391	1.00 (0.93, 1.07)	0.899	1.04 (1.03, 1.05)	<0.001	1.08 (0.89, 1.31)	0.454	1.09 (1.00, 1.20)	0.057	1.06 (1.01, 1.10)	0.012
**MDD**														
<Secondary	Ref.		Ref.		Ref.		Ref.		Ref.		Ref.		Ref.	
≥Secondary	1.04 (1.03, 1.42)	<0.001	1.03 (1.02, 1.05)	<0.001	1.11 (1.08, 1.14)	<0.001	1.04 (1.03, 1.04)	<0.001	1.07 (1.01, 1.15)	0.030	1.13 (1.09, 1.17)	<0.001	1.05 (1.02, 1.07)	<0.001
**MMF**														
<Secondary	Ref.		Ref.		Ref.		Ref.		Ref.		Ref.		Ref.	
≥Secondary	1.02 (1.016, 1.23)	<0.001	1.01 (1.00, 1.02)	0.157	1.05 (1.02, 1.09)	0.002	1.02 (1.01, 1.22)	<0.001	1.08 (1.02, 1.15)	0.008	1.06 (1.03, 1.10)	<0.001	1.03 (1.01, 1.04)	0.010
**MAD**														
<Secondary	Ref.		Ref.		Ref.		Ref.		Ref.		Ref.		Ref.	
≥Secondary	1.042 (1.037, 1.047)	<0.001	1.04 (1.02, 1.06)	<0.001	1.11 (1.09, 1.14)	<0.001	1.04 (1.03, 1.05)	<0.001	1.12 (1.05, 1.19)	<0.001	1.13 (1.09, 1.17)	<0.001	1.04 (1.02, 1.06)	<0.001
**CIRF**														
<Secondary	Ref.		Ref.		Ref.		Ref.		Ref.		Ref.		Ref.	
≥Secondary	1.03 (1.02, 1.04)	<0.001	1.02 (1.01, 1.04)	0.004	1.09 (1.06, 1.12)	<0.001	1.021 (1.016, 1.025)	<0.001	1.03 (0.97, 1.11)	0.306	1.05 (1.02, 1.09)	0.001	1.04 (1.02, 1.06)	<0.001

AOR=Adjusted odds ratio; CI=confidence intervals; Adjusted odds ratios, 95% confidence intervals, and p-values were obtained from the log-binomial regression model using the generalized estimating equation. Models adjusted for child's age, child's sex, maternal age, place of residence, and wealth index. For pooled data, besides these factors, we adjust the model by survey year. EIBF=Early initiation of breastfeeding, EBF= Exclusive breastfeeding, CBF=Continued breastfeeding at 1 year, ISSSF=Introduction of solid semi-solid and soft foods, MDD=Minimum dietary diversity, MMF=Minimum meal frequency, MAD=Minimum acceptable diet, CIRF=Consumption of iron-rich or iron-fortified foods.
